# Synthesis and molecular docking studies of new aryl imeglimin derivatives as a potent antidiabetic agent in a diabetic zebrafish model

**DOI:** 10.1038/s41598-024-60206-3

**Published:** 2024-04-24

**Authors:** Aylin Khodakhah, Hassan Mohammadi, Sina Abdoli, Issa Zarei, Mahdie Palimi, Zeinab Ekhtiari, Meysam Talebi, Mahmood Biglar, Mohammad Reza Khorramizadeh, Massoud Amanlou

**Affiliations:** 1 Department of Medicinal Chemistry, Faculty of Pharmacy, Tehran University of Medical Sciences, Tehran, Iran; 2 Biosensor Research Center, Endocrinology and Metabolism Molecular-Cellular Sciences Institute, and Zebra Fish Core Facility (ZFIN ID: ZDB-LAB-190117-2), Endocrinology and Metabolism Research Institute, Tehran University of Medical Sciences, Tehran, Iran; 3 Drug Design and Development Research Center, The Institute of Pharmaceutical Sciences (TIPS), Tehran University of Medical Sciences, Tehran, Iran; 4 Experimental Medicine Research Center, Tehran University of Medical Sciences, Tehran, Iran

**Keywords:** Diabetes mellitus, 1,3,5-triazine, Metformin, Imeglimin, Zebrafish, Drug discovery, Medicinal chemistry, Pharmacology, Screening

## Abstract

Diabetes mellitus (DM) is a persistent, progressive, and multifaceted disease characterized by elevated blood glucose levels. Type 2 diabetes mellitus is associated with a relative deficit in insulin mainly due to beta cell dysfunction and peripheral insulin resistance. Metformin has been widely prescribed as a primary treatment option to address this condition. On the other hand, an emerging glucose-reducing agent known as imeglimin has garnered attention due to its similarity to metformin in terms of chemical structure. In this study, an innovative series of imeglimin derivatives, labeled **3(a–j)**, were synthesized through a one-step reaction involving an aldehyde and metformin. The chemical structures of these derivatives were thoroughly characterized using ESI–MS, 1H, and 13C NMR spectroscopy. In vivo tests on a zebrafish diabetic model were used to evaluate the efficacy of the synthesized compounds. All compounds **3(a–j)** showed significant antidiabetic effects. It is worth mentioning that compounds **3b** (FBS = 72.3 ± 7.2 mg/dL) and **3g** (FBS = 72.7 ± 4.3 mg/dL) have antidiabetic effects comparable to those of the standard drugs metformin (FBS = 74.0 ± 5.1 mg/dL) and imeglimin (82.3 ± 5.2 mg/dL). In addition, a docking study was performed to predict the possible interactions between the synthesized compounds and both SIRT1 and GSK-3β targets. The docking results were in good agreement with the experimental assay results.

## Introduction

Type 2 diabetes mellitus (T2DM) is characterized by a relative deficiency of insulin, primarily caused by beta cell dysfunction and external insulin resistance^1^. Genetic and environmental factors, such as obesity and sedentary behaviors, also play a role in its development^2,3^. The International Diabetes Federation (IDF) predicts that diabetes will affect around 700 million people worldwide in the next two decades, leading to a significant economic impact due to its rising incidence and prevalence^4,5^. The cornerstone of treating T2DM lies in lifestyle modifications, including maintaining a healthy weight, engaging in regular exercise, and adopting a balanced diet^6^. Subsequently, implementing effective medical treatments becomes essential in improving the quality of life and reducing mortality rates for individuals living with diabetes^7^. Diabetes substantially elevates the risk of developing cardiovascular disease, kidney failure, blindness, neuropathy, and premature mortality^7,8^.

Metformin, an antidiabetic agent, was introduced in the United Kingdom in 1958 and received approval from the United States Food and Drug Administration in 1994^9,10^. Belonging to the class of drugs known as biguanides, metformin serves as the first-line treatment option for T2DM, effectively reducing liver glucose production and increasing body insulin sensitivity^11^. Notably, metformin exhibits favorable pharmacokinetic properties and demonstrates beneficial effects on cardiovascular health without causing hypoglycemia^9^. However, its derivatives, phenformin, and buformin, were once used as antidiabetic medications but were later withdrawn in many countries due to their association with an increased risk of lactic acidosis^9^.

1,3,5-triazine is well-known aromatic six-membered heterocyclic moiety and shows several biological and pharmacological properties. 1,3,5-triazines has a broad spectrum of biological activities including antibacterial^12^, antifungal^10^, antimalarial^13^, anticancer^12,14^, antiviral^15^, anti-inflammatory^16^, and antitubercular properties^17^.

Imeglimin is the first novel drug of the glimin class containing 1,3,5-triazine core which is synthesized from metformin^18^. The drug has been acting via various mechanisms, including targeting mitochondria bioenergetics, increasing insulin secretion, and protecting the endothelial or beta cells' death from oxidative stress^19^. Moreover, it demonstrated beneficial effects on the pancreas, liver, and skeletal muscles, which are adversely affected by diabetes^5^.

According to previous studies, several drugs that are used to treat the T2DM condition are often associated with various side effects, such as abdominal pain, hepatotoxicity, diarrhea, flatulence, and hypoglycemia^20^. Therefore, owing to their lack of safety, unsatisfactory efficacy, and cytotoxicity, further investigation for new antidiabetic agents is necessary.

Although imeglimin has not yet been approved by the United States Food and Drug Administration (FDA), imeglimin may provide a valuable new therapeutic option in the future. In fact, imeglimin has been shown to be more effective than metformin in various cases and has the potential to be used in various diseases^17,19,21^.

**Figure 1:**
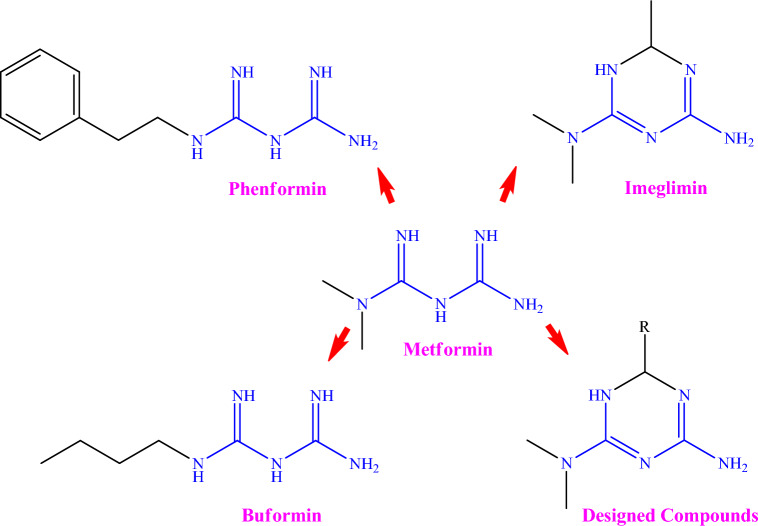
Chemical structure of some metformin derivatives and designed compounds.

Therefore, imeglimin derivatives deserve further studies, especially in terms of anti-diabetic properties. In exploring the potential of this approach based on the distinct and specific pharmacological advantages of the imeglimin backbone (1,3,5-triazine scaffold), we prepared several novel and potent imeglimin derivatives by replacing methyl of imeglimin with different aromatic substituents and evaluated their antidiabetic activity on zebrafish diabetic model in vivo (Fig. 1).

## Results and discussions

### Chemistry

**Scheme 1:**
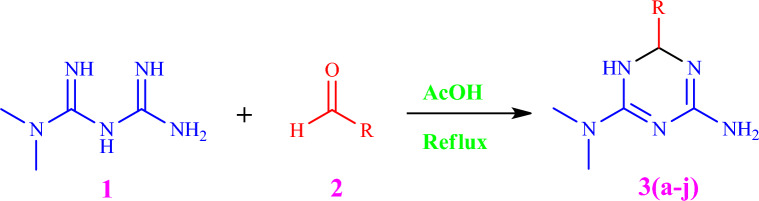
General Scheme for the synthesis of imeglimin derivatives **3(a–j)**.

Despite the introduction of numerous novel antidiabetic compounds in the pharmaceutical market, metformin continues to be extensively prescribed as the first-line pharmacotherapy owing to its distinct and favorable attributes. Recently, imeglimin, akin to metformin as a antihyperglycemic agent, has emerged as one of these new compounds^21^. As part of this study, ten innovative compounds were synthesized to serve as potential antidiabetic agents, following the pathway illustrated in Scheme 1.

To create these novel imeglimin derivatives, we adopted a one-pot reaction involving diverse aromatic aldehyde derivatives and metformin, serving as the biguanide component. The reaction was carried out using acetic acid as the solvent and under reflux conditions for a duration of 24 h, resulting in the successful synthesis of the compounds **3(a–j)**.

To purify the reaction mixture the precipitate obtained after evaporation of acetic acid, was washed several times with chloroform to remove excess aldehyde until a pure product was obtained. The yields of final compounds ranged from 69 to 88%, indicating a satisfactory outcome.

To characterize the synthesized compounds, their structures were confirmed through the utilization of ESI–MS and NMR spectra. Mass spectra data from the positive ionization (ESI +) mode was used for confirmation molecular weight of syntheszied compounds and in ESI spectra, peaks corresponding the (M + H)^+^ were observed for all final compounds. All synthesized compounds displayed ^1^H NMR and ^13^C NMR spectra consistent with the assigned structure.

### Antidiabetic activity

The anti-diabetic properties of the imeglimin derivatives **3(a–j)** were investigated on the zebrafish diabetic model at a concentration of 10 μM for 48 h. Metformin and imeglimin were used as the positive control (reference drugs). The results presented in Table 1 show that the synthesized compounds (**3b**, **3e**, and **3g**) showed promising anti-diabetic effects, surpassing those of imeglimin and metformin.

**Table 1:** Effect of the compounds **3(a–j)** on the FBS of zebrafish diabetic model**.**


Entry	R	M.W	FBS (mg/dl)
**3a**		217.28	92.0 ± 2.0
**3b**		260.35	72.3 ± 7.2
**3c**		231.26	94.7 ± 7.7
**3d**		247.30	80.3 ± 3.0
**3e**		262.27	78.7 ± 6.2
**3f.**		207.24	82.3 ± 10.7
**3g**		216.25	72.7 ± 4.3
**3h**		235.27	108.3 ± 11.0
**3i**		261.29	92.0 ± 6.9
**3j**		233.28	83.3 ± 3.3
Metformin*	–	129.17	74.0 ± 5.1
Imeglimin*	–	155.21	82.3 ± 5.2
Diabetic zebrafish	–	–	153.3 ± 15.5
Control	–	–	75.7 ± 4.4

*Metformin and imeglimin were used as standard references. Full results of statistical analysis performed and presented in supplementary file.

Metformin and imiglimin significantly decreased fatsing blood sugar (FBS) in diabetic zebrafish (*p* < 0.0001). However, these two drugs did not change the FBS levels of non-diabetic zebrafish (*p* > 0.999). In addition, FBS reduction in the metformin and imiglimin groups was not statistically significant (*p* = 0.991).

All compounds synthesized in this study were able to reduce FBS in diabetic zebrafish (*p* < 0.0001). Compounds **3b** (72.3 ± 7.2 mg/dL) and **3h** (108.3 ± 11.0 mg/dL) had the strongest and weakest ability to reduce FBS in diabetic zebrafish, respectively. Compounds **3g** and **3e** were considered among the strongest compounds after compound **3b**, respectively. With the exception of compound **3h**, which could not reduce the FBS of zebrafish to that of non-diabetic zebrafish, other synthesized compounds were able to reduce FBS to similar level of non-diabetic zebrafish (*p* > 0.3–0.99). These three compounds (**3b**, **3e**, and **3g**) demonstrated FBS levels comparable to those of the reference compounds metformin (74.0 ± 5.1 mg/dL) and imeglimin (82.3 ± 5.2 mg/dL). On the other hand, compounds **3c** and **3h** showed less efficacy compared to the reference drugs and other synthesized compounds (Supplementary material Figs. S1& S2).

Structure–activity relationship (SAR) studies suggest that the presence of electron-donating groups at the benzyl rings is crucial for maximizing potency (Table 1). However, in the case of compound **3c**, the hydroxyl substitution at the ortho-position of the phenyl ring likely attenuated the potency due to intramolecular hydrogen bonding with the triazine core. Surprisingly, compound **3g** (2-pyridine analog) and compound **3b** (with para-*N*,*N*-dimethylaminophenyl group) exhibited noteworthy antidiabetic properties and were considered as the most potent compounds in this series. These two compounds showed similar efficacy to metformin and were significantly more effective than imeglimin. The antidiabetic potential of compounds **3b** and **3g** may be attributed to the lone pair interaction of the respective nitrogen in the para-*N*,*N*-dimethylaminophenyl or pyridine moieties. In comparison to metformin, most of the imeglimin derivatives **3(a–j)** demonstrated acceptable anti-diabetic activities, with compounds **3b** and **3g** standing out as the most effective derivatives. The triazine core exhibited remarkable antidiabetic activities, sparking interest in further structure modifications as potential lead compounds for future investigations.

### Docking simulation method

There is no single protein, universally acknowledged as a valid target, responsible for biguanide’s bioactivities. In view of the wide variety of effects of these compounds, it is reasonable to assume that there is more than one pathway involved in their pharmacology. In addition, these imeglimin derivatives have a different effect compared to other anti-diabetic agents such as sulfonylureas and meglitinides, which work by modifying a single target.

Two proteins have been considered responsible for biguanide’s bioactivity in past computational studies^22,23^. Sirtuin 1 (SIRT1) is a novel target that has been introduced more recently than glycogen synthase kinase-3 beta (GSK-3β). SIRT1 regulates glucose/lipid metabolism through its deacetylase activity on many substrates. SIRT1 in pancreatic β cells positively regulates insulin secretion, protects cells from oxidative stress and inflammation, and modulates insulin signaling in the metabolic pathway. It has been proposed that biguanides such as metformin have a positive regulatory activity on SIRT1^22^. GSK-3B has long been considered to be responsible for metformin bioactivity^23^. GSK-3β is a key enzyme in glycogen synthesis and plays a key role in the regulation of blood glucose. More importantly, GSK-3β is one of the key factors leading to insulin deficiency and insulin resistance. Inhibitors of GSK-3β have been shown to increase insulin sensitivity, glycogen synthesis, and glucose metabolism in skeletal muscles of diabetic patients^23^.

We performed molecular docking simulations to investigate the binding modes and possible interactions of the synthesized compounds **3(a–j)** with the active sites of the SIRT1 and GSK-3β targets and compared them to the binding energies of metformin and imeglimin. Docking validation was done by re-docking the co-crystal ligands of the crystal structures of 1Q4L and 5BTR, and the RMSD values were lower than 2 Å, indicating the accuracy of the docking method.

Three compounds **3b**, **3e**, and **3g**, which showed high antidiabetic activity against the zebrafish diabetic model, were selected for docking studies to determine the possible interaction with SIRT1 and GSK-3β. Table 2 lists the docking binding energies (kcal/mol) predicted by molecular docking.

**Table 2:** FBS and docking binding energy of compounds **3b**, **3e**, and **3g** with SIRT1 and GSK-3β.

Entry	FBS (mg/dl)	Binding energy* (GSK-3β)	Binding energy* (SIRT1)
3b	72.3 ± 7.2	− 7.7	− 7.3
3e	78.7 ± 6.2	− 7.5	− 7.2
3g	72.7 ± 4.3	− 7.2	− 6.9
Metformin	74.0 ± 5.1	− 4.4	− 4.5
Imeglimin	82.3 ± 5.2	− 5.2	− 5.5

*(kcal/mol).

**Figure 2:**
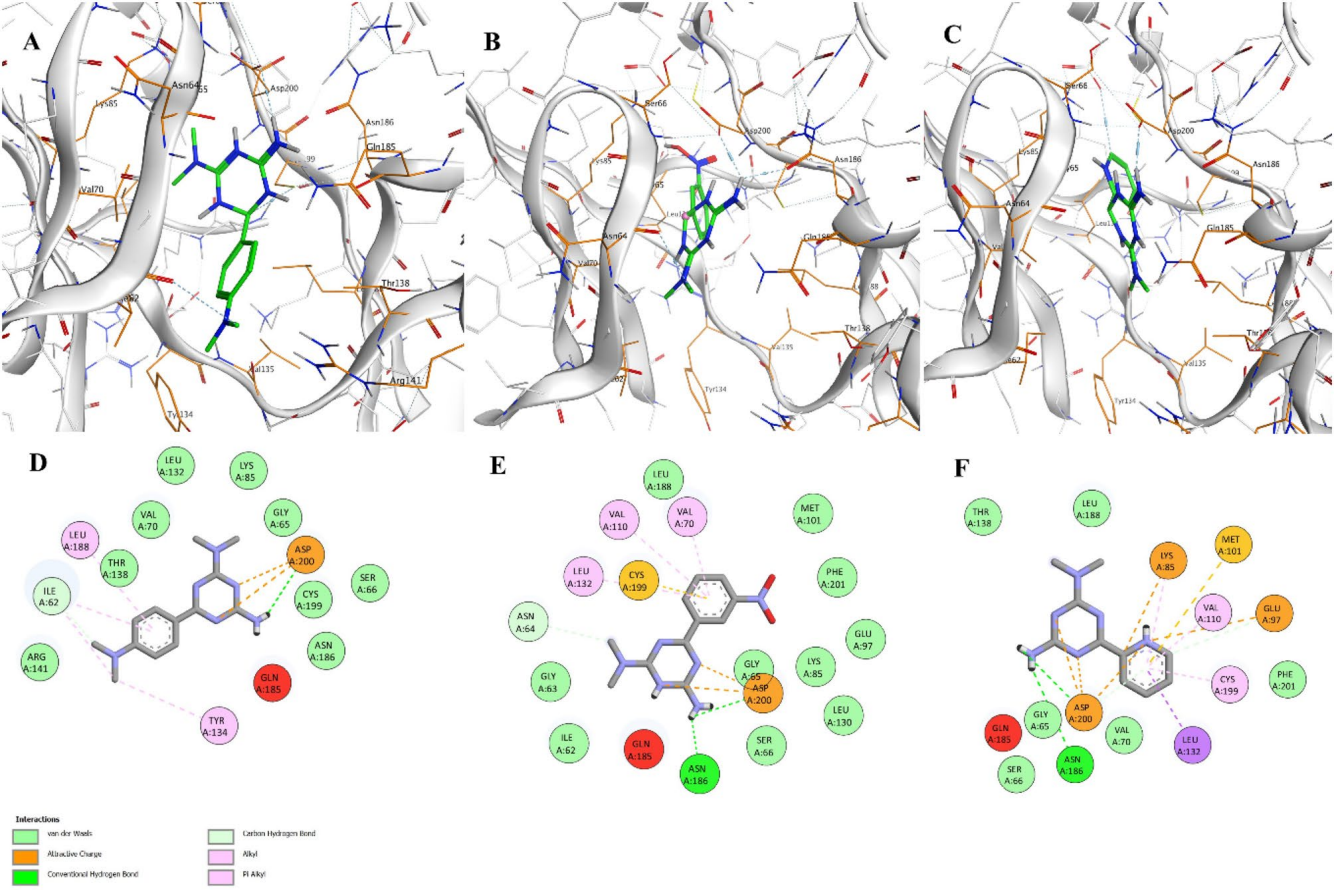
Ligand binding interactions in the active site of GSK-3β, (**A**) **3b**, (**B**) **3e**, (**C**) **3g**. and 2D display of the interactions of compounds **D**) **3b**, (**E**) **3e**, (**F**) **3g**.

The docking results of GSK-3β indicate that Leu188, Gln185, and Asp200, with the same bond type, have alkyl–pi interactions, with the free amine at the end of the biguanides, and Asp200 with an attractive charge, interact with all three compounds 3b, 3e, and 3g. The superior docking binding energy of 3b in the active site of GSK-3β may be related to the specific volume of the para-N,N-dimethylaminophenyl moiety, which is easily occupied and positioned in the active site by the two methyl groups (Fig. 2A–F).

**Figure 3:**
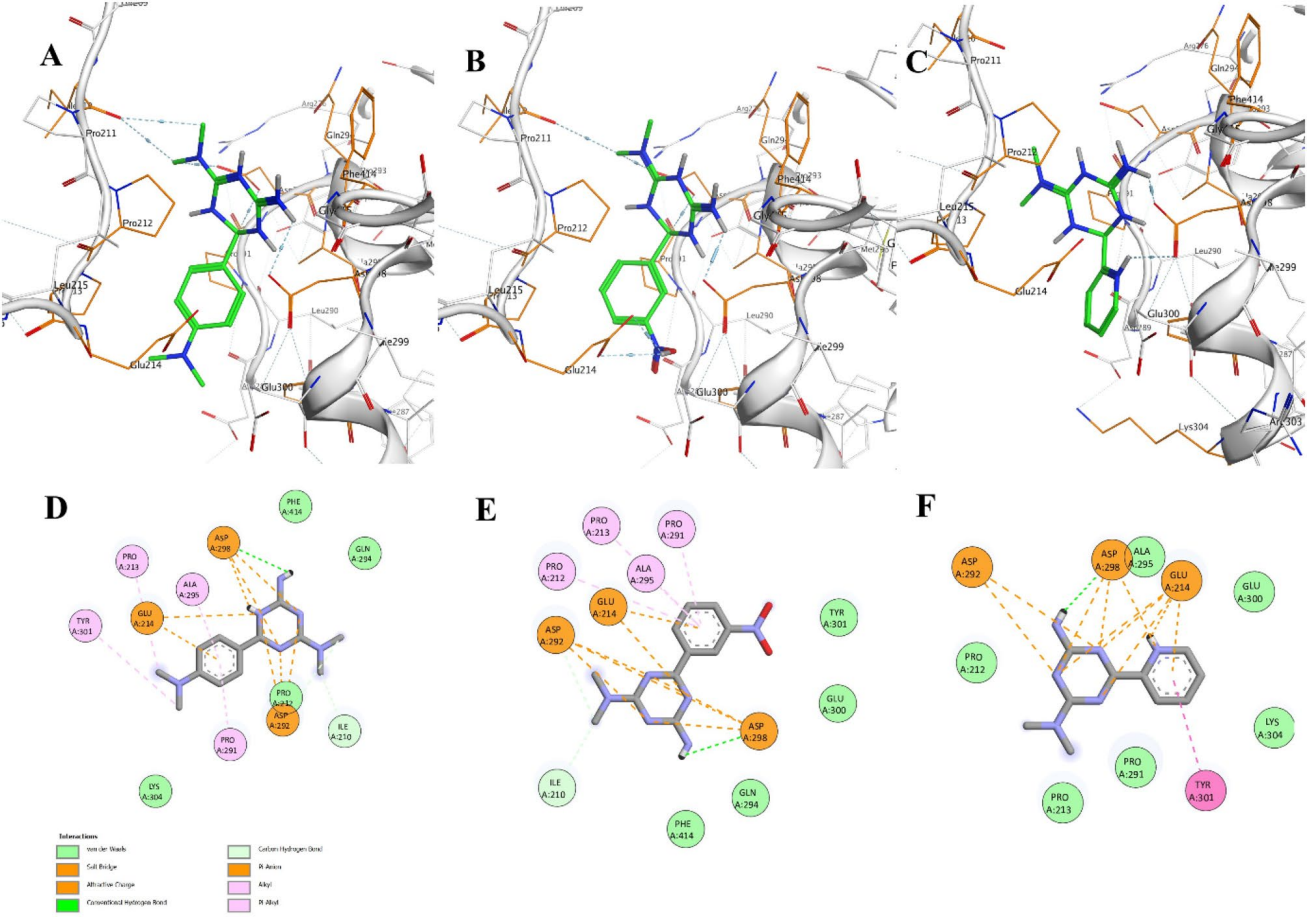
Ligand binding interactions in the active site of SIRT1, (**A**) **3b**, (**B**) **3e**, (**C**) **3g**. and 2D display of the interactions of compounds (**D**) **3b**, (**E**) **3e**, (**F**) **3g**.

The docking results of SIRT1 and Ala295, Pro213, and Pro291 with alkyl-pi interactions were the same as those of Asp298 and Asp292 with biguanides, and the electrostatic attractive charge bonds of Asp298 and Asp292 with biguanides were the same as those between SIRT1 and compounds 3b, 3e, and 3g. However, the highest binding energy between 3b and SIRT1 may be related to a more favorable van der Waals interaction (Fig. 3A–F).

**Figure 4:**
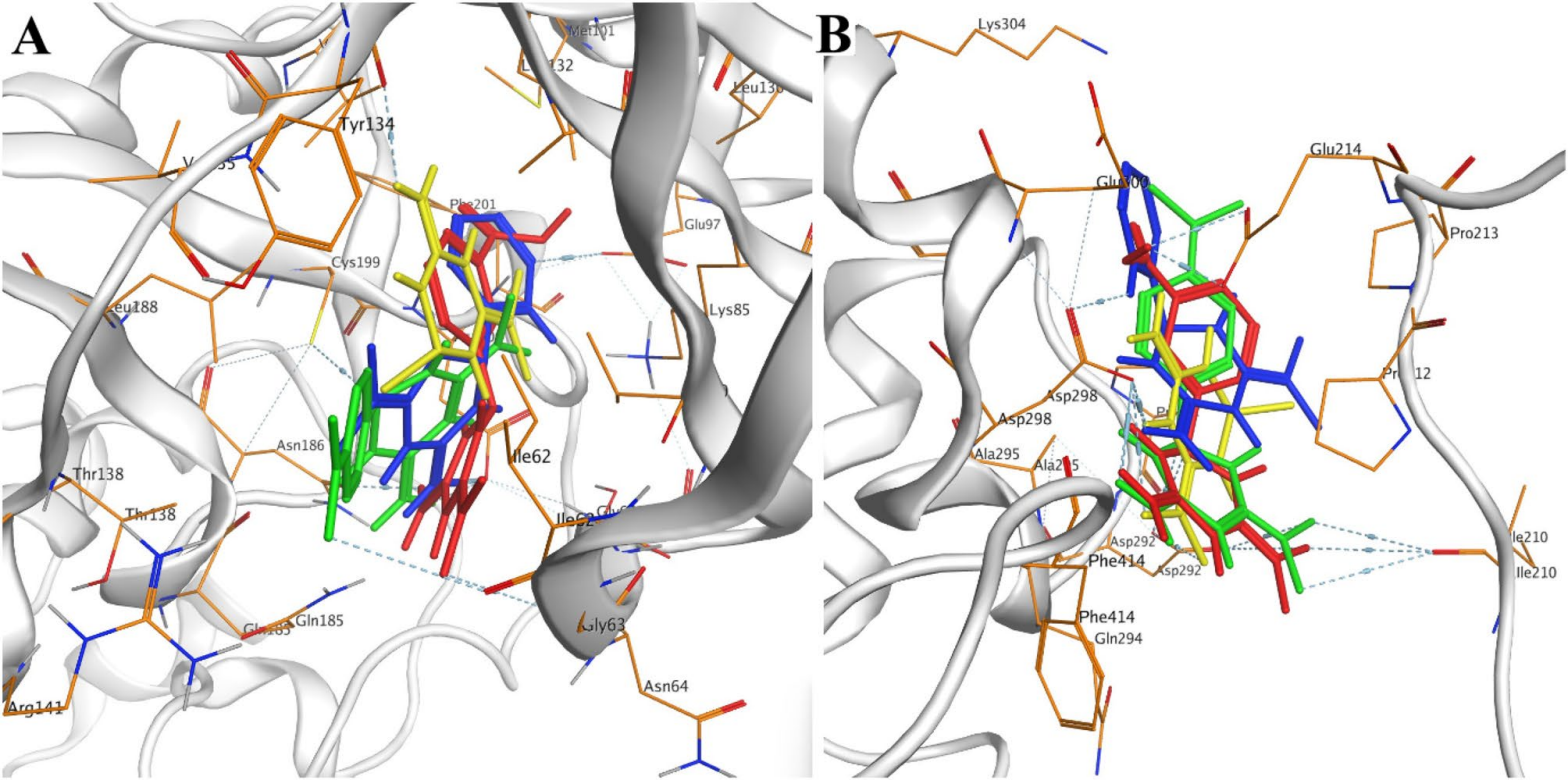
Superimpose position of compounds **3b** (green), **3e** (red), **3g** (blue) and imeglimin (yellow) in the active site of (**A**) GSK-3β and (**B**) SIRT1.

The superimposed positions of the most potent compounds **3b**,** 3e**, and **3g** in the active sites of the SIRT1 and GSK-3β indicate that all compounds are well positioned inside the cavity with proper orientation (Fig. 4).

Despite the slight difference between the docking energies for compounds **3b**, **3e**, and **3g**, there is a qualitative agreement between the FBS and docking binding energies for both SIRT1 and GSK-3β targets and all three ligands are good options for further consideration.

## Conclusion

In summary, we designed and synthesized 10 new imeglimin analogs, labeled **3(a–j)**, and evaluated their antidiabetic effects on a zebrafish diabetic model. Some of these compounds demonstrated significant anti-diabetic activity and showed greater efficacy than standard metformin. The synthesized derivatives, **3(a–j)**, were characterized by 1H-NMR, 13C-NMR, and ESI–MS techniques. The two compounds **3b** (72.3 ± 7.2 mg/dL) and **3g** (72.7 ± 4.3 mg/dL), as the strongest compounds in this study, have the same potential as metformin in reducing FBS (74.0 ± 5.1 mg/dL).

The structure–activity relationship (SAR) study highlighted the significance of electron-donating groups on the aryl moiety of the target compounds in enhancing their antidiabetic activity. The remarkable anti-diabetic potential of compounds 3b and 3g warrants further investigation of their efficacy and safety, making them promising candidates for the development of more effective and safer antidiabetic drugs. These findings provide valuable insights for future research endeavors aimed at advancing diabetes treatment options.

## Experimental

### General methods

All the chemicals used in this study were procured from renowned sources, namely Merck and Sigma-Aldrich. These chemicals were used without any further purification. A Kofler hot stage apparatus was used to determine the melting points. The 1H and 13C NMR spectra were recorded using a Bruker FT-500 instrument with TMS as the internal standard. Chemical shifts (δ values) are expressed in ppm relative to internal solvent peaks, and coupling constants (J) are measured in Hertz. Signals are described as s (singlet), d (doublet), t (triplet), m (multiplet), and brs (broad singlet).

Reaction completion and purity of the synthesized compounds were verified using TLC on silica gel 250-μm F254 plastic sheets. The mass spectra were acquired using the SCIEX Triple Quad 5500 + LC–MS/MS instrument.

### Docking method

Crystallographic structures of biguanide receptors with PDB ID: 1Q4L and 5BTR were obtained from a protein data bank (www.rcsb.org) for studying the bioactivity of compounds 3(a–j) on GSK-3β and SIRT1. These two structures were chosen because they already contain experimentally studied ligands; thus, choosing a grid box would be easier and more accurate. All water molecules, ligands, and ions were removed from the PDB file for the preparation of the input file. Polar hydrogens were then added, and the Kollman-united charge was used to calculate the partial atomic charge. The prepared file was saved in pdbqt format and used in the following steps. 3D structures of three target compounds and two controls were generated and energy minimized by the MM2 force field using Chem3D 12 and saved in PDB format. OpenBabel (version 2.3.1) was used to convert PDB to PDBQT. Data were prepared, and docking was performed by AutoDock Vina 1.2.3^24^.

A 40 × 40 × 40 Å (x, y, z) grid box dimension with 0.375 nm spacing was centered in the reported pocket of each protein reported in previous studies for GSK-3β and SIT1^22,23^. For GSK-3β, the grid box was centered in 40.118, 4.584, 34.328 (x, y, z), and for SIRT1, the grid box was centered in 21.647 52.885 12.831 (x, y, z), and the exhaustiveness was set at 8. Discovery Studio Visualizer version 17.2 and PyMol version 1.1 visualized the docking results. The docking results were visualized by Discovery Studio Visualizer version 17.2 and PyMol version 1.1.

### Zebrafish assay

#### Ethical statement

All zebrafish experiments were conducted according to the standard animal guidelines approved by the Animal Care Committee of the Tehran University of Medical Sciences and Endocrinology and Metabolism Research Center (IR.TUMS.VCR.REC.1395.8, IR.TUMS.AEC.1401.060). In addition, all procedures for animal experiments described in this study were performed in accordance with the care and use of laboratory animals and ARRIVE guidelines.

### Conditioning of adult Zebrafish

Three-month-old zebrafish (*Danio rerio*) with a body length of 2.0–2.5 cm was randomly chosen and divided into fourteen groups (a diabetic model group, two positive control groups, a negative group, and 10 experimental groups) and transferred into 6-well plates so that one fish placed in each well (20 ml). The fish plates were kept at constant filtration, continuous air conditioning, pH around 7, and a temperature of 28 ± 1 °C. They were then set aside in a photoperiod of 16 h of light, and 8 h of darkness daily and fed once a day with 7% of their body weight^25^. The average weight per fish was 3g. Each group experiment was repeated for three times.

### Blood glucose measerment

On the last day of treatment, the fish were fasted for 24 h for blood sampling and blood glucose measurement. After blood sampling, a glucometer was used to measure the blood glucose levels. In biological studies, anesthesia in animals is usually carried out using substances approved and studied to ensure animal welfare. The clove powder extract (250 mg/L) was used for fish anesthesia in the present study. Several steps have been taken before blood sampling. In order to avoid potential side effects, it is necessary to prepare an appropriate concentration of clove extract and to know the correct dosage. Fish were placed in an incubator containing 250 mg clove powder extract in 1 L of water. Parameters, such as heart rate, respiration, and overall condition, were monitored in order to ensure the welfare of animals. After complete anesthesia by cutting the caudal fins, a glucometer was used to measure the blood glucose level in the fish.

### Hyperglycemia induction

The Hyperglycemia modeling process induces a diabetic state in fish subjects for research. For acclimation (induction of glucose) gradually increasing glucose concentrations of 50, 100, and 200 mM were applied for 10, 7, and one day respectively^25^. To maintain accuracy, glucose solutions were exchanged every 12 h, ensuring sufficient oxygen supply, and preventing contamination. Fish were closely monitored for stress and movement issues, and fed daily. A control group without added glucose provided a comparison. This approach enhances diabetes research, providing valuable insights and potential therapeutic implications.

### Hypoglycemic effect determination on Zebrafish diabetic model

In the zebrafish experimental groups, compounds **3(a–j)** (10 µM) and metformin hydrochloride (10 µM) were administered for 48 h, and the culture medium was refreshed once. Subsequently, the culture medium was replaced with fresh water to eliminate glucose interference during the sampling process. Blood glucose levels were measured using a Match blood sugar test device. The fish's posterior tail was cut using tweezers, and the removed blood was immediately brought into contact with the test strip to obtain the fasting blood sugar (FBS) readings^26^.

### General procedure for the synthesis of aryl 1,3,5-triazine derivatives 3(a–j)

Metformin (5 mmol) was dissolved in glacial acetic acid (35 ml) as a solvent to prepare the imeglimin derivatives. Subsequently, various aldehyde derivatives (5.5 mmol) were carefully added dropwise to the reaction medium. The reaction mixture was then refluxed overnight in an oil bath at 108 °C with continuous stirring. The progress of the reaction was periodically monitored using TLC. After completion of the reaction, the resulting mixture was evaporated under vacuum. Finally, the mixture was washed three times with chloroform to eliminate any excess aldehyde to obtain the desired and pure compounds **3(a–j)**. The successful synthesis of the imeglimin derivatives for further investigation is ensured by this process.

#### *N*^*2*^*,N*^*2*^-dimethyl-6-phenyl-1,6-dihydro-1,3,5-triazine-2,4-diamine (3a)

White powder; yield: 88%; m.p.: 257–259 °C; ^1^H NMR (500 MHz, D_2_O): δ (ppm) 7.60–7.31 (m, 5H, Ar), 5.83 (q, *J* = 5.2 Hz, 1H, N–CH–N), 3.08 (S, 3H, Me), 3.04 (S, 3H, Me); ^13^C NMR (125 MHz, D_2_O): δ (ppm) 157.77, 156.21, 138.97, 129.93, 129.41, 126.31, 63.14, 23.28; LRMS (ESI +): m/z: calcd for C_11_H_15_N_5_ + H^+^:218.13 [M + H]^+^, found: 218.13.

#### 6-(4-(dimethylamino)phenyl)-*N*^*2*^*,N*^*2*^-dimethyl-1,6-dihydro-1,3,5-triazine-2,4-diamine (3b)

Brown powder; yield: 72%; m.p.: 287–289 °C; ^1^H NMR (300 MHz, DMSO-d6): δ (ppm) 9.65 (1H, NH), 8.17 (d, *J* = 15.72 Hz, 4H, Ar), 2.52 (s, 6H, 2 × NCH3), 1.63 (s, 6H, 2 × NCH3); **HRMS (ESI +)**: m/z: calcd for C_13_H_20_N_6_/2 + H^+^ + K^+^:150.07 [M/2 + H + K]^2+^, found: 150.00.

#### 2-(4-amino-6-(dimethylamino)- 1,3,5-triazin-2-yl)phenol (3c)

White powder; yield: 69%; m.p.: 257–259 °C; ^1^H NMR (300 MHz, DMSO-d6) δ(ppm) 7.29–6.80 (m, 4H, Ar), 2.10 (s, 6H, 2 × NCH3); LRMS (ESI +): m/z: calcd for C_11_H_13_N_5_O + H^+^:232.11 [M + H]^+^, found: 232.08.

#### 6-(2-methoxyphenyl)-* N*^*2*^*,N*^*2*^-dimethyl-1,6-dihydro-1,3,5-triazine-2,4-diamine (3d)

White powder; yield: 79%; m.p.: 210–213 °C; ^1^H NMR (500 MHz, D_2_O): δ (ppm) 7.37–7.25 (m, 1H, Ar), 7.11 (dd, *J* = 7.6, 1.7 Hz, 1H, Ar), 6.99 (d, *J* = 8.3 Hz, 1H, Ar), 6.91 (t, *J* = 7.5 Hz, 1H, Ar), 5.97 (s, 1H, N–CH–N), 3.78 (s, 3H, OCH_3_), 2.93 (s, 6H, 2 × NCH_3_); ^13^C NMR (125 MHz, D_2_O) δ(ppm) 157.43, 156.58, 155.98, 130.92, 126.46, 125.70, 120.59, 111.75, 59.07, 55.53, 36.89, 27.45; LRMS (ESI +): m/z: calcd for C_12_H_17_N_5_O + H^+^:248.14 [M + H]^+^, found: 248.07.

#### *N*^*2*^*,N*^*2*^-dimethyl-6-(3-nitrophenyl)-1,6-dihydro-1,3,5-triazine-2,4-diamine (3e)

White powder; yield: 79%; m.p.: 210–213 °C; ^1^H NMR (500 MHz, D_2_O): δ (ppm) 8.17–8.12 (m, 2H, Ar), 7.74 (d, *J* = 7.9 Hz, 1H, Ar), 7.58 (t, *J* = 7.9 Hz, 1H, Ar), 5.91 (s, 1H, N–CH–N), 3.02–2.94 (m, 6H, 2 × Me); ^13^C NMR (125 MHz, D_2_O) δ(ppm) 157.37, 155.87, 148.04, 140.82, 132.73, 130.44, 124.42, 121.09, 61.97, 37.17, 36.54; LRMS (ESI +): m/z: calcd for C_11_H_14_N_6_O_2_ + H^+^:263.12 [M + H]^+^, found: 263.08.

#### 6-(furan-2-yl)-* N*^*2*^*,N*^*2*^-dimethyl-1,6-dihydro-1,3,5-triazine-2,4-diamine (3f)

White powder; yield: 86%; m.p.: 198–201 °C; ^1^H NMR (500 MHz, D_2_O): δ (ppm) 7.49 (s, 1H, Ar), 6.56–6.16 (m, 2H, Ar), 5.89 (d, *J* = 2.8 Hz, 1H, N–CH–N), 3.00 (d, *J* = 2.8 Hz, 6H, 2 × Me); ^13^C NMR (125 MHz, D_2_O) δ(ppm) 160.02, 156.12, 151.13, 144.39, 110.82, 108.29, 57.12, 37.74, 36.78; LRMS (ESI +): m/z: calcd for C_9_H_13_N_5_O + H^+^:208.11 [M + H]^+^, found: 208.07.

#### *N*^*2*^*,N*^*2*^-dimethyl-6-(pyridin-2-yl)-1,3,5-triazine-2,4-diamine (3g)

Brown powder; yield: 75%; m.p.: 292–296 °C; ^1^H NMR (500 MHz, DMSO-*d*_6_): δ (ppm) 8.56 (s, 1H, Ar), 8.15 (d, *J* = 8.4 Hz, 1H, Ar), 7.99 (t, *J* = 7.3 Hz, 1H, Ar), 7.67 (s, 1H, Ar), 3.11 (S, 3H, Me), 2.99 (S, 3H, Me); ^13^C NMR (125 MHz, D_2_O): δ(ppm) 161.85, 159.42, 158.06, 148.19, 145.29, 140.54, 129.33, 124.18, 37.04; LRMS (ESI +): m/z: calcd for C_10_H_12_N_6_ + H^+^:217.11 [M + H]^+^, found: 217.08.

#### 6-(4-fluorophenyl)-* N*^*2*^*,N*^*2*^-dimethyl-1,6-dihydro-1,3,5-triazine-2,4-diamine (3h)

White powder; yield: 67%; m.p.: 225–228 °C; ^1^H NMR (300 MHz, DMSO-d6): δ (ppm) 7.60 (m, 2H, Ar), 7.41 (m, 2H, Ar), 2.51 (s, 3H, Me), 0.76 (s, 3H, Me). LRMS (ESI +): m/z: calcd for C_11_H_14_FN_5_ + H^+^:236.12 [M + H]^+^, found: 236.07.

#### 2-(4-amino-6-(dimethylamino)-1,2-dihydro-1,3,5-triazin-2-yl)benzoic acid (3i)

White powder; yield: 78%; m.p.: 241–243 °C; ^1^H NMR (500 MHz, D_2_O): δ (ppm) 7.86 (S, 1H, Ar), 7.80 (d, *J* = 10.3 Hz, 2H, Ar), 7.66 (d, *J* = 14.7 Hz, 1H, Ar), 6.03–5.92 (m, 1H, N–CH–N), 3.22–3.16 (m, 3H, Me), 3.15 – 3.07 (m, 3H, Me); ^13^C NMR (125 MHz, D_2_O) δ(ppm) 166.36, 157.58, 152.93, 139.36, 135.85, 131.47, 128.94, 125.52, 124.78, 65.06, 38.42, 37.60; LRMS (ESI +): m/z: calcd for C_12_H_15_N_5_O_2_-OH^-^:244.12 [M-OH]^+^, found: 244.08.

#### 4-(4-amino-6-(dimethylamino)-1,2-dihydro-1,3,5-triazine-2-yl)phenol (3j)

White powder; yield: 71%; m.p.: 246–247 °C; ^1^H NMR (500 MHz, D_2_O): δ (ppm) 7.24 (d, 2H, Ar), 6.86 (d, 2H, Ar), 5.90–5.56 (m, 1H, N–CH–N), 3.04 (s, 6H, 2 × Me); ^13^C NMR (125 MHz, D_2_O) δ(ppm) 157.76, 156.89, 156.25, 130.66, 128.23, 115.99, 63.02, 37.41, 36.68; LRMS (ESI +): m/z: calcd for C_11_H_15_N_5_O + H^+^:234.13 [M + H]^+^, found: 234.08.

### Supplementary Information

Supplementary Figures.

## Data Availability

The all data necessary for confirming the conclusions presented in the article are represented fully within the article.
