# Supplementary material for: Synthesis and molecular docking studies of new aryl imeglimin derivatives as a potent antidiabetic agent in a diabetic zebrafish model
Source: Sci Rep. 2024 Apr 24;14:9410. doi: 10.1038/s41598-024-60206-3 (PMC11043428; doi:10.1038/s41598-024-60206-3)
Supplement: Supplementary file 1 — Supplementary Figures. [file 41598_2024_60206_MOESM1_ESM.docx]

**Figure 1s.** Histograms represent the effect of treatment with imeglimin, metformin, and newly synthesized derivatives (3a-3j) on fasting blood glucose (FBG) levels (mg/dL) in the diabetic and control zebrafish groups, respectively. The data were analyzed using one-way analysis of variance followed by Welch’s t-test compared with diabetic zebrafish (*p < 0.05). Each column represents the means ± SEM (n = 3).

**Figure 2s.** Histograms represent the effect of treatment with imeglimin, metformin, and newly synthesized derivatives (3a-3j) on fasting blood glucose (FBG) levels (mg/dL) in the diabetic and control zebrafish groups, respectively. The data were analyzed using one-way analysis of variance followed by Welch’s t-test compared with metformin (*p < 0.05). Each column represents the means ± SEM (n = 3).
